# Label-free quantitative proteomics and stress responses in pigs—The case of short or long road transportation

**DOI:** 10.1371/journal.pone.0277950

**Published:** 2022-11-23

**Authors:** Alessio Di Luca, Andrea Ianni, Michael Henry, Camillo Martino, Paula Meleady, Giuseppe Martino

**Affiliations:** 1 Department of Bioscience and Agro-Food and Environmental Technology, University of Teramo, Teramo, Italy; 2 National Institute for Cellular Biotechnology, Dublin City University, Dublin, Ireland; 3 Istituto Zooprofilattico Sperimentale dell’Abruzzo e del Molise “Giuseppe Caporale”, Teramo, Italy; Swiss Institute of Bioinformatics, SWITZERLAND

## Abstract

Ethical livestock production is currently a major concern for consumers. In parallel, research has shown that transport duration is an important factor affecting animal welfare and has a negative impact on the final product quality and on the production cost. This study applied proteomics methods to the animal stress/welfare problem in pigs muscle-exudate with the aim to identify proteins indicative of molecular processes underpinning transport stress and to better characterise this species as a biomedical model. A broader perspective of the problem was obtained by applying label-free LC-MS to characterise the proteome response to transport stress (short or long road transportation) in pigs within the same genetic line. A total of 1,464 proteins were identified, following statistical analysis 66 proteins clearly separating pigs subject to short road transportation and pigs subject long road transportation. These proteins were mainly involved in cellular and metabolic processes. Catalase and stress-induced phosphoprotein-1 were further confirmed by Western blot as being involved in the process of self-protection of the cells in response to stress. This study provide an insight into the molecular processes that are involved in pig adaptability to transport stress and are a step-forward for the development of an objective evaluation method of stress in order to improve animal care and management in farm animals.

## Introduction

Animal welfare is a major consideration in animal productions, in order to produce safe and quality food. Over the last few years, the growing population worldwide, the increasing prosperity in the newly industrialising countries and changed pattern of food consumption is constantly increasing the demand for products of animal origin [1]. In order to tackle some of these issues, the animal production systems have been intensified in order to be more efficient and to increase production. Different housing strategies and management treatments have been investigated because of their influence on animal welfare, performance and product quality [2].

In spite of the growing interest in animal welfare, the assessment is not a simple issue and requires a multi-criteria approach. It has been accepted that a definition of animal welfare has to include the following elements: i) the emotional state of the animal, ii) its biological functioning and iii) its ability to show normal patterns of behaviour [3]. Road transportation of animals is a practice that animals encounter in the livestock industry affecting animal welfare and lastly meat quality in pigs [4, 5]. Pigs transportation to the abattoir or to another holding expose animals to a series of stressful events including poor animal handling, departure from the usual room, loading, variable climatic conditions, noise, vibration during the journey, mixing with unfamiliar animals, inappropriate stocking density and unloading at the farm or at the abattoir. Animal responses to these stressful situations include physiological changes and change of the behaviour that allow for a rapid recovery or adaptation to the change. A failure in the response to these factors could lead to serious consequences ranging from weight loss, injury of the animals, impaired carcass, low meat quality, up to the death of the animals [6, 7].

Stress is the condition in an animal that results from the action of one or more stressors that may be of either external or internal origin. One of the most accepted definitions of stress is: the biological response caused when an individual perceives a threat to its homeostasis [8]. Animal responsiveness to stress is different from animal to animal and, as it is very difficult to establish the status of the welfare of each individual, there is the interest in the development of reliable animal-based methodologies to assess the level of stress, the effect on the welfare and lastly the ultimate meat quality of the animal [2].

Proteomics could expand our current knowledge on the biochemical cellular events involved in the animal’s responsiveness to stress. In addition, proteomics could provide biomarkers that define differences in the response to a specific stress. Mass spectrometry (MS) -based technologies are powerful tools contributing to our understanding of the dynamics, interactions and roles played by the proteins, advancing our understanding of biology on a systems-wide level for a wide range of applications [9]. Label-free quantitative proteomics can measure peptides present in a sample and can subsequently determine the abundance of the proteins across various samples. This method has been shown to provide greater global proteome coverage than labelling strategies, but with a lower quantitative precision [10]. Moreover, it has the advantage that require a lower sample volume, the sample processing is straightforward and has the capacity for measuring multiple biomarkers simultaneously [11].

Very few proteomics studies have investigated the influence of pre-slaughter handling practices like animal transportation. For example, Morzel et al. [12], using 2D electrophoresis, investigated the influence of transport conditions on proteome changes of pork *longissimus dorsi* muscle. Eight spots were found to be differentially changes in abundance on the 2D gels, mitochondrial ATPase was characterised and was found to be overexpressed in the group transported immediately before slaughter. Using ELISA, Yu et al. [13] investigated the effect of transportation on the expression of four heat shock proteins (alpha-B-crystalline, Hsp27, Hsp70 and Hsp90) in the *longissimus dorsi* of pigs, observing a decreased abundance of these proteins after transportation.

In this study, we used label-free quantitative proteomics to conduct proteomic profiling of muscle exudate (diaphragm) collected following centrifugation from pigs after short or long road transport from the farm to the abattoir, which represented conditions of no stress and high degree of stress, respectively. Following statistical analyses, two proteins were further validated by Western blotting, with a view to identify proteins and peptides indicative of molecular processes underpinning transport stress.

## Materials and methods

All animals used in this study were housed according to the Italian and European legislations for pig production. Animals were electrically stunned and then slaughtered under controlled conditions in an EU licensed commercial abattoir located in the municipality of Castel Castagna, Teramo, Italy. This facility daily welcomes pigs of the same genetic type coming from farms of the northern Italy (about 3 hours; animals stressed for long road transportation) as well as animals from the farm attached to the slaughterhouse (about 15 minutes; animals non-stressed), for which transport is obviously reduced to a minimum. The animals subjected to the sampling were therefore randomly selected from these two categories indicated. For these reasons, the experimental treatments were not specifically designed for the purposes of this study and no farming practices other than those normally adopted have been introduced.

### Sampling

Eight commercial hybrid gilts from the same genetic type (Pig Improvement Company; PIC) were included in this study, of which four pigs followed a short road transportation (about 15 minutes; animals non-stressed) and the other four followed a long road transportation (about 3 hours; animals stressed for long road transportation) to the abattoir the day of slaughtering. All the animals used in this study were in a state of good health. The management of all pigs included in this study with regard to housing, feeding and handling was the same in order that the transport time was the only difference between both groups. Pigs from both groups were fed with the same standard commercial feed for fattening pigs under the production rules of the Parma and San Daniele dry-cured ham consortia. The experiment was carried out in autumn, with similar temperature in both animal house (ranged between 18 and 23°C), the outside temperatures were from 8 to 14°C at the morning of the transport day. Care was taken to avoid mixing unfamiliar pigs, each group of four pigs coming from the same rearing pen, was loaded in the same truck pen. All animals had been fasted overnight (before and during transport), both transports were carried out on the same day in the morning. On arrival at the slaughterhouse, each group was unloaded, electrically stunned and slaughtered within 30 min of arrival (non-stressed group was slaughtered at 08:00; stressed group was slaughtered at 09:15) under the same conditions at a live weight of approximately 155 ± 5 kg.

### Sample preparation for label-free LC-MS/MS analysis

Exudate was collected the same day of slaughtering from the diaphragm muscle following the modified protocols of Di Luca et al. [14] and Bouton, Harris, and Shorthose [15]. We reduced the centrifugation speed to 3400×g at 4°C and increased the amount of samples to three 10 g cubes (weight of each cube ~3.3 g; ~1 cm^3^) taken from the diaphragm muscle from each sample the day of slaughtering. Care was taken to avoid obvious pieces of fat. These were centrifuged in disposable polypropylene centrifuge tubes (50 ml; Thermo Fisher Scientific) for 60 min (Mega Star 3.0, VWR International Srl, IT). Following centrifugation, the exudate was snap frozen in liquid nitrogen and stored at -80°C until required. The amount of exudates collected using this method ranged from about 20 μl to 350 μl (average of about 80 μl). The protein concentration of all samples was determined in triplicate using the Bio-Rad Protein Assay Kit (Bio-Rad Labs, Hercules, CA, USA), following the Bradford method using a BSA standard and were found to be between 70 and 86 μg/μl [16].

Four biological replicate (one sample from each animal) for each group (stressed and non-stressed) were subject to label-free analysis. Equal amounts (100 μg) of all protein samples were reduced and alkylated with dithiothreitol (DTT) and iodoacetamide (IAA; Sigma-Aldrich/Merck, USA) and then digested with trypsin according to the FASP method [17]. This is a commonly used method to purify and digest protein samples that has been successfully applied to the analysis of different type of samples, providing highly reproducible protein abundance values that improve sensitivity, recovery and proteomic coverage for processed samples. To achieve this, FASP use an ultrafiltration device with a membrane pores that allows detergents to pass through, while proteins are retained because of their bigger size [18]. Peptides were then purified using C18 spin columns (Thermo Fisher Scientific), dried under vacuum and suspended in 2% acetonitrile and 0.1% trifluoroacetic acid prior to mass spectrometry analysis.

### LC-MS/MS and label-free quantitative differential analysis

Liquid chromatography-mass spectrometry (LC-MS/MS) was performed on a Dionex (USA) UltiMate3000 nanoRSLC coupled in-line with an Orbitrap Fusion Tribrid™ mass spectrometer (Thermo Scientific). Briefly, one μl of digest was loaded onto the trapping column (PepMap100, C18, 300 μm × 5 mm, 5 μm particle size, 100 Ǻ pore size;Thermo Scientific) for 3 minutes at a flow rate of 25 μL/min with 2% (v/v) ACN, 0.1% (v/v) TFA. Peptides were resolved on an analytical column [Acclaim PepMap 100, 75 μm × 50 cm, 3 μm bead diameter column; (Thermo Scientific)] using the following binary gradient; solvent A (0.1% (v/v) formic acid in LC-MS grade water) and solvent B (80% (v/v) ACN, 0.08% (v/v) formic acid in LC-MS grade water) using 2–32% B for 75 minutes, 32–90% B in 5 minutes and holding at 90% for 5 minutes at a flow rate of 300 nL/min.

MS1 spectra were acquired over m/z 380–1500 in the Orbitrap (120 K resolution at 200 m/z), and automatic gain control (AGC) was set to accumulate 4 × 10^5^ ions with a maximum injection time of 50 ms. Data-dependent tandem MS analysis was performed using a top-speed approach (cycle time of 3 s), with precursor ions selected in the Quadrupole with an isolation width of 1.6 Da. The intensity threshold for fragmentation was set to 5000 and included charge states 2+ to 7+. Precursor ions were fragmented using Higher energy Collision Dissociation (HCD) with a normalized collision energy of 28% and the MS2 spectra were acquired with a fixed first m/z of 110 in the ion trap. A dynamic exclusion of 50 s was applied with a mass tolerance of 10 ppm. AGC was set to 2 × 10^4^ with a maximum injection time set at 35 ms.

Protein identification was achieved using Proteome Discoverer v.2.2 (Thermo Fisher Scientific) with the Sequest HT algorithm and Percolator. MS files were searched against *Sus Scrofa* protein database from UniProt (1,428 reviewed proteins and 47,760 unreviewed TrEMBL) downloaded April 2019. The following search parameters were set for protein identifications: (i) MS/MS mass tolerance set at 0.6 Da; (ii) peptide mass tolerance set to 10 ppm; (iii) up to two missed cleavages were allowed; (iv) cysteine carbamidomethylation set as a static modification; (v) methionine oxidation set as a dynamic modification. Only highly confident peptide identifications with a false discovery rate (FDR) ≤ 0.01 were considered (identified using a Sequest HT workflow coupled with Percolator validation in Proteome Discoverer 2.2).

Quantitative label-free analysis was carried out using Progenesis QI for Proteomics software version 2.0 (NonLinear Dynamics, UK), as recommended by the manufacturer (see www.nonlinear.com for further background to alignment, normalisation, calculation of peptide abundance, etc.). As already described elsewhere [19], the software extracts quantitative information from MS1 data by aligning the data based on the LC retention time of each sample to a reference file (sample run that yielded most peptide ions). This allow for any drift in retention time, giving an adjusted retention time for all runs in the analysis. Results were filtered, based on statistical analysis. The Progenesis peptide quantification algorithm calculates peptide abundance as the sum of the peak areas within its isotope boundaries. Each abundance value is then transformed to a normalised abundance value by applying a global scaling factor. Protein abundance was calculated as the sum of the abundances of all peptide ions which have been identified as coming from the same protein. Only peptide ions with charge states +1, +2 and + 3 were allowed and normalized. The normalized peptide abundance data was transformed prior to statistical analysis, using an arcsinh transformation to meet the assumptions of the one-way ANOVA test. Peptides with a one-way ANOVA p value ≤0.05 between experimental groups were exported and identified using Proteome Discoverer as described. Protein identifications were imported into Progenesis QI for proteomics and considered differentially expressed proteins if they passed the following criteria: (i) proteins with ≥2 peptides matched, (ii) a ≥1.5 fold difference in abundance and (iii) an ANOVA between experimental groups of ≤0.05.

### Western blot

Two proteins [stress induced phosphoprotein 1 (STIP1) and catalase (CAT)] that had been found regulated by label-free LC/MS were selected for confirmation using Western blot. Muscle exudate samples from four biological replicate from the non-stressed and stressed pigs (one sample from each animal, for a total of eight animal samples) were separated on a 12% Bis-Tris SDS PAGE gel, loaded and transferred onto 0.2 μm PVDF membranes (Bio-Rad). To increase the accuracy of the confirmation the Western blot analysis was repeated three times using the same eight biological replicates. Twenty micrograms of proteins were loaded in each lane for the samples that were later incubated with the antibody mouse monoclonal antibody stress induced phosphoprotein 1 (STIP1) raised against amino acids 203–453 mapping near the C-terminus of STI1 of human origin (Santa Cruz, USA, sc-390203) whereas, 25 micrograms of proteins were loaded in each lane for the samples that were later incubated with the antibody mouse monoclonal antibody catalase (H-9) mapping between amino acids 471–503 near the C-terminus of catalase of mouse origin (Santa Cruz, USA, sc-271803). To ensure successful transfer of proteins and to allow for accurate quantitation of proteins load, membranes were stained using the reversible stain Ponceau S (Sigma-Aldrich). Images of the stained membranes were then acquired using Azure Biosystems C400 (USA). The stain was then removed using water, then blocked with 5% non-fat milk and then incubated overnight with the primary antibodies (2–8°C) with a concentration of 1:500 for both proteins STIP1 and CAT. The membranes were then incubated with the secondary antibodies for 1 h. For all primary antibodies, the secondary antibody used was polyclonal donkey anti-mouse IgG HPR conjugated (1:2500, SA1-100, Thermo Fisher Scientific). Membranes were finally subjected to electrochemiluminescent detection using Westar ŋC Ultra 2.0 (Cyanagen, IT), acquired (Azure Biosystems C400, USA) and analysed using Image J software [20]. The average band density was then normalised to the average band density of the lane to control for any loading inaccuracies [21]. The statistical analysis of the normalised average band density of STIP1 and CAT proteins was carried out across the two phenotypes using ANOVA and Tukey’s test on R [22]. Phenotype was included as a fixed effect.

### Bioinformatics

Proteins that were detected as statistically differentially abundant between pigs were submitted to classification analysis using the PANTHER (Protein Analysis Through Evolutionary Relationships) database system, release 14.1 (http://www.pantherdb.org/) [23]. Default parameters were used to carry out the analysis. Data were acquired for two functional classifications proposed by PANTHER: biological process and pathway.

The STRING (Search Tool for the Retrieval of Interacting Genes/Proteins) database (https://string-db.org/; version 11) was used to carry out the in silico Protein-Protein Interaction (PPI) analysis of the 30 differentially expressed proteins up-regulated in the non-stressed pigs and of the 36 proteins that were up-regulated in stressed pigs [24]. The analyses were carried out considering the *Sus scrofa* specific interactome. Only interactions having a STRING combined score > 0.4 (medium confidence) were considered. Network indices such as the number of nodes and edges, the average node degree (average no. of connections), the expected number of edges and the PPI enrichment p-value were computed. The expected number of edges gives how many edges would be expected if the nodes were selected at random. A small PPI enrichment p-value indicates that nodes are not random and that the observed number of edges is significant.

## Results

### Proteomic analysis of pig muscle exudate after short or long road transportation

Muscle exudate samples collected *post mortem* (day 0) from four biological replicated from non-stressed (short road transportation) and stressed (long road transportation) pigs were processed using label-free proteomic analysis.

The analysis of the non-stressed pig samples by Proteome Discoverer peptide and protein identification identified a total of 6,169 peptides belonging to 1,283 proteins, whereas the analysis in the stressed pig samples identified 6,102 peptides related to 1,227 proteins. A total of 1,464 proteins were identified combining these two datasets (S1 Table). The majority of these proteins were mainly involved in cellular process (35.8%), in metabolic process (24.5%), biological regulation (12.3%) and localisation (7.9%) following PANTHER analysis.

### Comparative proteomic analysis of pig muscle exudate between short and long road transportation

Differences at the proteome level between the group of pigs subject to short road transportation (non-stressed) and the group of pigs subject to long road transportation (stressed) were investigated by using the software incorporated in Progenesis QI for proteomics. Proteins were ranked by *p* value derived from one-way ANOVA (p ≤ 0.05), fold change (≥1.5) and the number of peptides (≥2) matched to the protein.

Label-free data analysis showed 66 proteins vary significantly in abundance between the non-stressed pigs compared to the stressed pigs. Of these, 30 proteins demonstrated an increased abundance and 36 a decreased abundance in the non-stressed pigs. Details of the significant variation between phenotypes are presented in Table 1.

**Table 1:** 66 proteins identified with altered levels in response to transport stress (stressed) compared to short road transportation (non-stressed) following label-free MS/MS analysis (Progenesis QI for proteomics).

UniProt^a)^	Gene Name	Identification	Peptides^b^	Score^c^	Anova (p)	Fold change	Highest condition^d^
A5YV76	FASN	Fatty acid synthase	2	7.01	0.002	177.74	NON-STRESSED
A0A287AFQ4	C8G	Complement C8 gamma chain	2	7.86	0.011	3.41	NON-STRESSED
A0A287AQ20	CFI	Complement factor I isoform 1 preproprotein	2	7.25	0.001	12.46	NON-STRESSED
A0A287A042	MGAM	Maltase-glucoamylase	2	5.21	0.004	2.39	NON-STRESSED
A0A287A217	TUBB4B	Tubulin beta chain	2	7.23	0.042	5.05	NON-STRESSED
F1RG45	AGT	Angiotensinogen	2	6.88	0.009	1.97	NON-STRESSED
A0A287B3S3	ITIH1	Inter-alpha-trypsin inhibitor heavy chain H1	2	5.35	0.012	2.4	NON-STRESSED
Q2HYU1	CKMT2	Creatine kinase, mitochondrial 2	2	5.63	0.006	2.9	NON-STRESSED
F1S8Y0	ANKRD2	Ankyrin repeat domain 2	2	9.19	0.009	2.1	NON-STRESSED
F1RZN7	KLKB1	Kallikrein B1	2	6.80	0.018	2.91	NON-STRESSED
A0A287AIM8	C5	Complement C5a anaphylatoxin	2	5.52	0.002	3.52	NON-STRESSED
F1S0N2	ACLY	ATP-citrate synthase	2	6.08	0.005	11.1	NON-STRESSED
F1SMN5	FLNC	Filamin C	2	6.17	0.022	4.94	NON-STRESSED
F1S564	RTCA	RNA 3’-terminal phosphate cyclase	2	6.38	0.028	18.75	NON-STRESSED
A0A286ZVF6	LOC102161784	GB1/RHD3-type G domain-containing protein	2	7.35	0.046	7.91	NON-STRESSED
F1SMJ1	C7	Complement component C7	2	5.73	0.013	3.32	NON-STRESSED
F1SB81	PLG	Plasminogen	2	7.52	0.044	3.24	NON-STRESSED
I3L9N5	PSMD5	Proteasome 26S subunit, non-ATPase 5	2	6.03	0.002	3.63	NON-STRESSED
A0A287AG58	PZP	Pregnancy zone protein	2	7.03	0.020	2	NON-STRESSED
A0A287BF33	ACTA1	Actin, alpha skeletal muscle	2	8.96	0.011	1.6	NON-STRESSED
F1RKY2	SERPIND1	Serpin family D member 1	2	6.00	0.002	2.35	NON-STRESSED
B3STX9	F2	Prothrombin	3	8.04	0.011	2.86	NON-STRESSED
L8B180	IGHG	IgG heavy chain	3	5.87	0.003	2.55	NON-STRESSED
K7GND8	CLU	Clusterin	3	7.77	0.015	2.37	NON-STRESSED
F1S0J2	C4BPA	C4b-binding protein alpha chain	3	10.97	0.013	3.11	NON-STRESSED
A0A286ZJK1	CFH	Complement factor H	3	8.47	0.008	3.38	NON-STRESSED
A0A287BM29	APOA4	Apolipoprotein A-IV	4	13.86	0.004	5.44	NON-STRESSED
B3CL06	TF	Transferrin	6	20.84	0.048	2.24	NON-STRESSED
F1SBS4	C3	Complement C3	7	19.98	0.005	2.84	NON-STRESSED
A0A287BAY9	ALB	Serum albumin	7	25.83	0.014	2.84	NON-STRESSED
I3LHC8	BDH2	3-hydroxybutyrate dehydrogenase 2	2	6.21	0.005	3.64	STRESSED
F1SK00	IDH2	Isocitrate dehydrogenase [NADP]	2	4.26	0.001	2.5	STRESSED
A0A287B8G0	PKM	Pyruvate kinase	2	6.70	0.012	2.66	STRESSED
I3LP02	ACAT1	Acetyl-CoA acetyltransferase 1	2	10.28	0.022	4.44	STRESSED
F1SKY2	NIT2	Nitrilase family member 2	2	6.34	0.007	2.15	STRESSED
A0A286ZUK9	PPP1R16A	Protein phosphatase 1 regulatory subunit 16A	2	4.87	0.008	3.1	STRESSED
A0A287AGG3	PSMB5	Proteasome subunit beta	2	6.71	0.021	3.05	STRESSED
I3LRS5	ALDH1A1	Aldehyde dehydrogenase 1 family member A1	2	5.61	0.012	2.73	STRESSED
A0A287AT21	SORD	Sorbitol dehydrogenase	2	9.86	0.014	2.61	STRESSED
I3L9I6	BCAM	Basal cell adhesion molecule (Lutheran blood group)	2	4.91	0.016	2.68	STRESSED
A0A287AMP3	CKM	Creatine kinase M-type	2	9.64	0.001	2.32	STRESSED
F1RPV1	TIPRL	TOR signaling pathway regulator	2	6.02	0.017	2.14	STRESSED
F1S814	PGM1	Phosphoglucomutase 1	2	9.28	0.013	3.62	STRESSED
A0A2C9F380	PGM3	Phosphoacetylglucosamine mutase	2	5.68	0.001	2.55	STRESSED
A0A287BDV9	GPD1	Glycerol-3-phosphate dehydrogenase [NAD(+)]	2	5.44	0.005	2.2	STRESSED
F1SIX3	UGP2	UTP—glucose-1-phosphate uridylyltransferase	2	6.09	0.026	2.47	STRESSED
F2Z5L7	PSMA1	Proteasome subunit alpha type	2	6.38	0.021	2.33	STRESSED
A0A286ZX40	ACY1	Aminoacylase-1	2	4.15	0.002	3.13	STRESSED
A0A287B088	PSMB4	Proteasome subunit beta	2	6.59	0.016	3.21	STRESSED
A0A287BIV4	PSMA4	Proteasome subunit alpha type	2	5.64	0.006	3.27	STRESSED
F1S9Q3	HSPA8	Heat shock cognate 71 kDa protein	3	8.57	0.013	2.11	STRESSED
A0A287B4J2	AHCY	Adenosylhomocysteinase	3	9.55	0.016	2.9	STRESSED
I3LNG8	STIP1	Stress induced phosphoprotein 1	3	7.11	0.027	2.31	STRESSED
Q9N0M7	CAPN1	Micromolar calcium-activated neutral protease 1 isoform B	4	12.88	0.013	2	STRESSED
F1SGS9	CAT	Catalase	4	14.79	0.023	2.64	STRESSED
F1SV14	RDX	Radixin	4	12.73	0.026	2.13	STRESSED
Q8WMK2	CKM	Muscle creatine kinase (Fragment)	4	8.50	0.006	2.49	STRESSED
F1RRD6	PDCD6IP	BRO1 domain-containing protein	5	13.52	0.006	2.4	STRESSED
A0A287BG23	GAPDH	Glyceraldehyde-3-phosphate dehydrogenase	5	12.08	0.001	2.71	STRESSED
A0A287A1V5	ALDOA	Fructose-bisphosphate aldolase	5	16.05	0.018	4.9	STRESSED
E0D7H7	AmpT	Leukotriene A(4) hydrolase	5	13.39	0.005	2.17	STRESSED
F1RQQ8	PYGM	Alpha-1,4 glucan phosphorylase	6	17.22	0.001	2.2	STRESSED
A0A287B3F4	AGL	Amylo-alpha-1, 6-glucosidase, 4-alpha-glucanotransferase	6	19.79	0.010	2.51	STRESSED
F1SRI8	MYBPC1	Myosin binding protein C, slow type	7	18.59	0.001	1.72	STRESSED
F1RPH0	PGK1	Phosphoglycerate kinase	7	21.96	0.006	2.55	STRESSED
A0A2C9F393	VCL	Vinculin	8	24.85	0.002	2.25	STRESSED

30 proteins were up-regulated in the non-stressed pigs (Table 1) and 36 proteins were up-regulated in stressed pigs (Table 1).

^a^) Accession number in the UniProt database.

^b^) Peptides used for quantitation.

^c^) SEQUEST score.

^d^) Indicates if the proteins were up-regulated in the non-stressed or in the stressed pigs.

### Functional inference of proteomic differences between animals transport length

**Fig 1:**
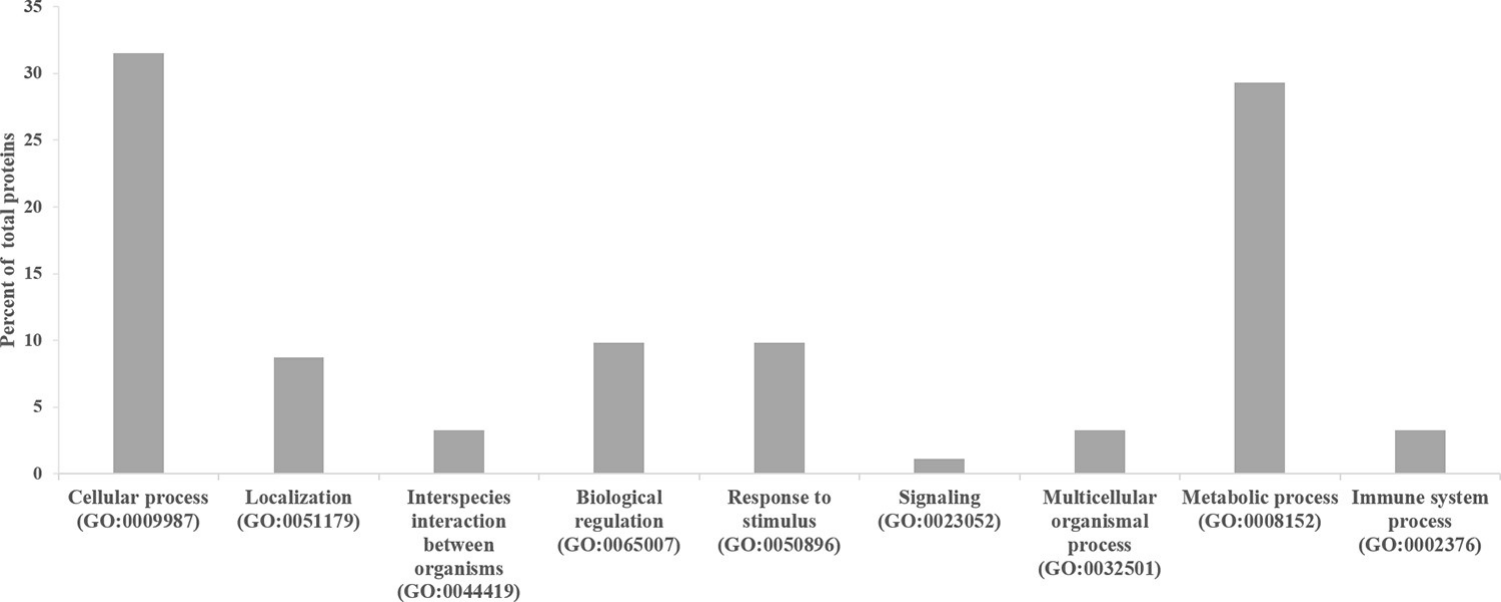
PANTHER database classification of the 66 proteins differentially expressed (Table 1). Proteins were classified according to their biological processes.

All the 66 proteins differentially expressed that were identified were submitted to classification analysis using PANTHER online database in order to obtain further information about the biological processes and pathways in which these proteins are involved. Considering the GO classification for biological processes, all the differentially abundant proteins were divided in nine classes such as cellular process, localization, interspecies interaction between organisms, biological regulation, response to stimulus, signaling, multicellular organismal process, metabolic process and immune system process, two of which (cellular process and metabolic process) accounted each for more than 29% of the total listed proteins (Fig 1). These proteins were involved in 20 pathways (S1 Fig), the majority of which were involved in blood coagulation (12%) huntingon disease (12%) and glycolysis (12%).

**Fig 2:**
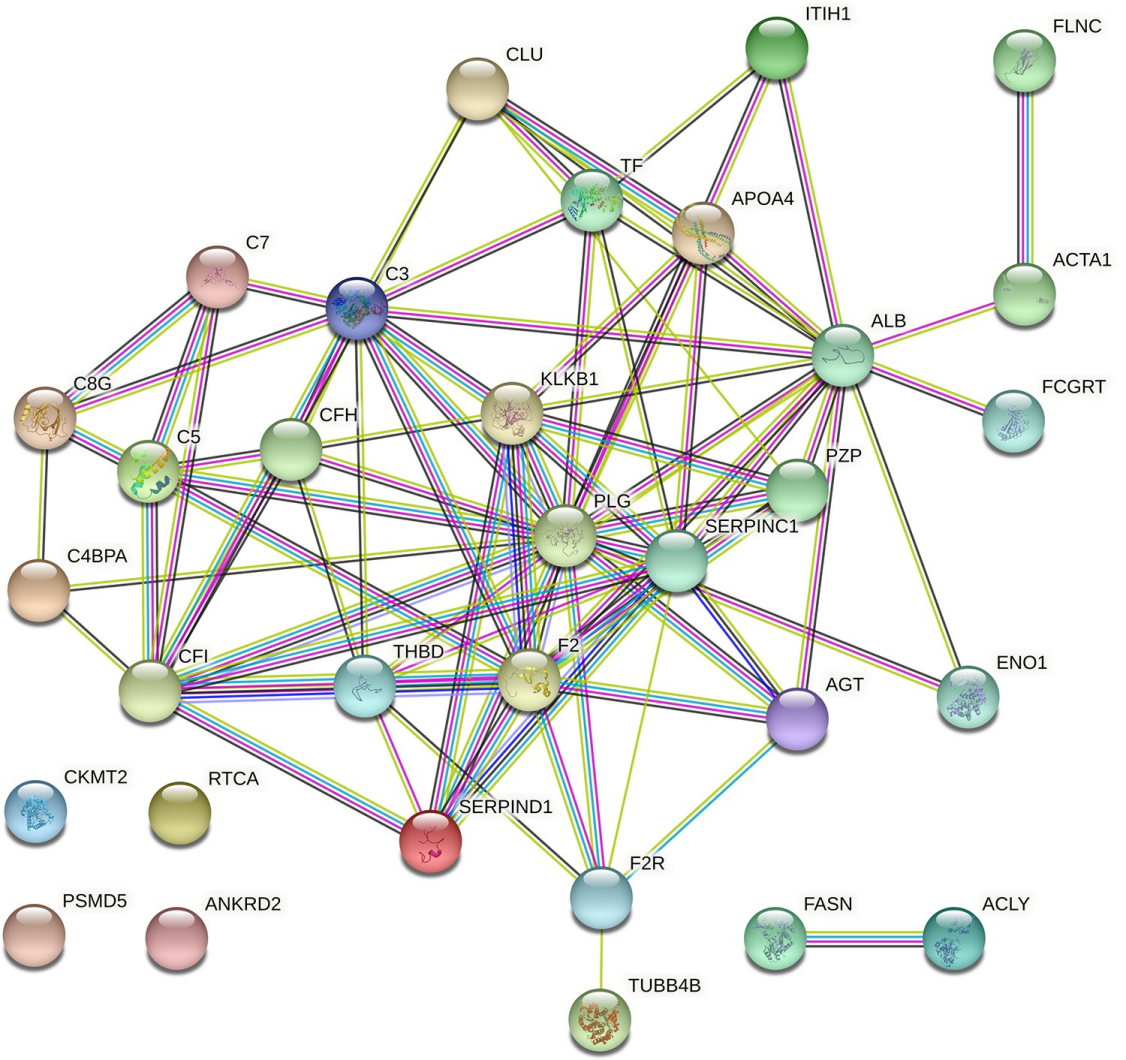
Protein-protein interaction map of the 30 proteins differentially expressed up-regulated in the non-stressed pigs. Interactions are based on STRING v.11.0 software analysing the identified proteins against *Sus scrofa* database. Interactions are shown in different colors: cyan is from curated databases, magenta is experimentally determined, dark green is gene neighbourhood, red is gene fusion, blue is gene co-occurrence, light green is textmining, black is co-expression and light blue is protein homology.

**Fig 3:**
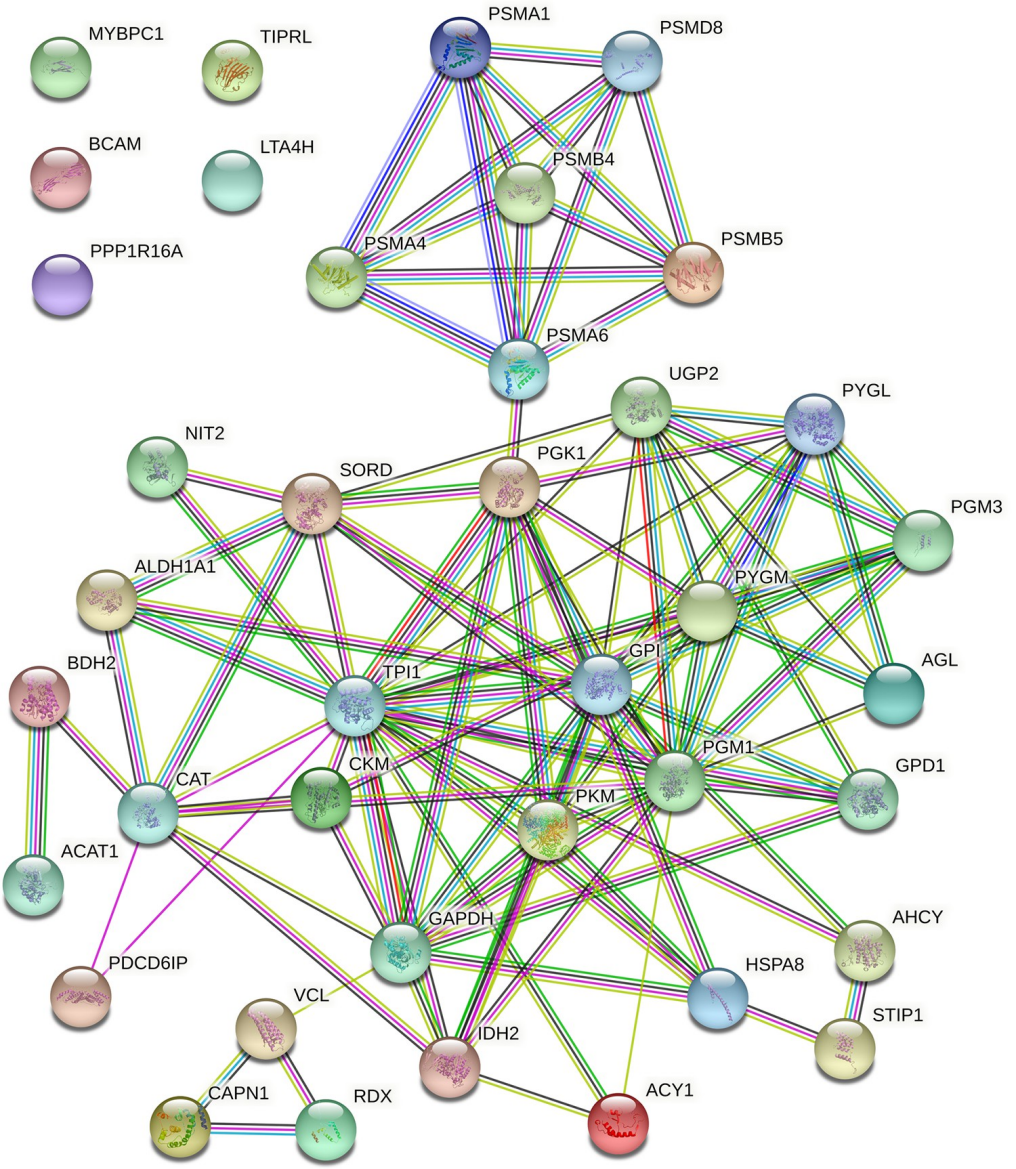
Protein-protein interaction map of the 36 proteins differentially expressed up-regulated in the stressed pigs. Interactions are based on STRING v.11.0 software analysing the identified proteins against *Sus scrofa* database. Interactions are shown in different colors: cyan is from curated databases, magenta is experimentally determined, dark green is gene neighbourhood, red is gene fusion, blue is gene co-occurrence, light green is textmining, black is co-expression and light blue is protein homology.

STRING was used to highlight the functional connections established among the 30 differentially expressed proteins up-regulated in the non-stressed pigs and among the 36 proteins that were up-regulated in the stressed pigs. The analysis of the 30 differentially expressed proteins up-regulated in the non-stressed pigs revealed a connected protein network composed by one big module composed by 32 nodes (Fig 2) divided in: one big module composed by 26 nodes (81.25%), a small component of two proteins (6.25%) and 4 singletons (12.5%). The resulting network showed a protein-protein interaction enrichment p-value of < 1.0e-16 (13 expected edges vs. 85 detected edges) indicating that proteins are at least partially biologically connected, as a group. Most of the proteins in this network interacted with five or six other partners (average node degree equal to 5.31). The analysis of the 36 proteins that were up-regulated in the stressed pigs revealed a connected protein network composed by one big module composed by 39 nodes (Fig 3) divided in: one big module composed by 34 nodes (87.18%) and 5 singletons (12.82%). The resulting network showed a protein-protein interaction enrichment p-value of < 1.0e-16 (21 expected edges vs. 105 detected edges) indicating that proteins are at least partially biologically connected, as a group. Most of the proteins in this network interacted with five or six other partners (average node degree equal to 5.38). In addition, from the big module there is a cluster of six proteasome proteins; these proteins in the label-free analysis were more abundant in the animals subject to long road transportation.

### Confirmation of differential protein expression using Western blot

**Fig 4:**
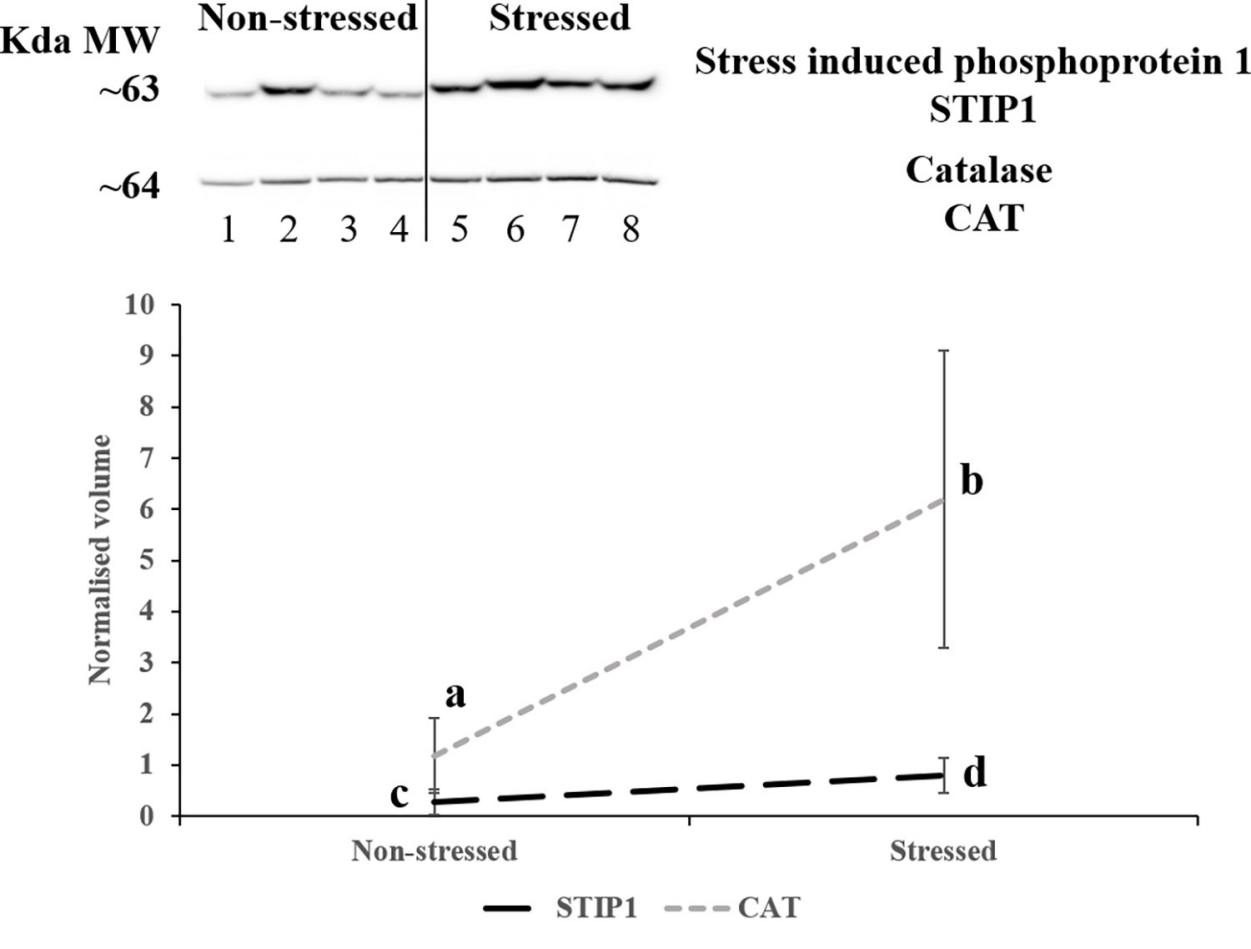
Western blot of stress induced phosphoprotein 1 (STIP1) and catalase (CAT) in pig muscle exudate. Means are derived from four biological replicates used for each phenotype. Numbers (1 to 8) at the bottom of the image indicate the eight animals used in the experiment for each pig (1 to 4: non-stressed pigs; 5 to 8: stressed pigs), each of which was run in an individual gel lane. Three technical replicates were run for each animal and the normalised value was used for statistical analysis. The graph shows the normalised average band density of STIP1 and CAT across the two phenotypes, superscripts show which phenotype are significantly different at p<0.05 [a and b for CAT (grey dots) and c and d for STIP1 (black segments)].

Western blot was used to confirm the abundance pattern of two proteins [stress induced phosphoprotein 1 (STIP1) and catalase (CAT)] among the 66 proteins differentially expressed that were identified. Three technical replicates were analysed for each sample. The average of the normalised band density of the three technical replicates was used for statistical comparison. Fig 4 shows representative images for STIP1 and CAT proteins. Both proteins, following label-free proteomics, were more abundant in the animals subject to long road transportation (stressed), and Western blot analysis confirmed the pattern observed by label-free proteomics. This is shown quantitatively in the graph in Fig 4, which presents the abundance pattern of STIP1 and CAT.

## Discussion

Animal welfare problems are important for ethical reasons and because they may cause great economic losses. However, there is not a standard procedure to evaluate with accuracy the degree of animal welfare and the level of stress of an animal [25, 26]. In this study, we used label-free LC-MS proteomics to the animal stress/welfare problem, with the aim to provide a more detailed picture of the pathways and processes underpinning transport stress in pigs. To achieve this we characterised the proteomic response to transport stress in muscle exudate from pigs after short or long road transport from pigs that are commonly used in the pig industry. In our study, we compared the proteome differences of two group of samples, after few minutes of transport (non-stressed animals) and after 3 hours of transport (stressed animals), thanks to the technological advancements of the mass spectrometer used have resulted in substantial improvements in proteome characterisation after 3 hours of transport [27]. Indeed, this approach allowed us to identify 66 proteins (36 up-regulated and 30 down-regulated in the animals subjected to long road transportation) that appear to be strongly involved in transport stress response. Two proteins, catalase and stress induced phosphoprotein 1 were further validated by Western blot.

The use of direct behavioural observations (e.g. using behaviour recognition video) and of biomarkers that can reflect the pathophysiological responses to stress are the most common methodologies used to evaluate stress in animals [28–30]. Nevertheless, objective criteria to evaluate animal stress needs to be improved. Indeed, monitoring methods of animal behaviour is labour intensive and involves subjective errors [31], whereas conventional biomarkers are often weak in their specificity and sensitivity [32]. For example, cortisol is the most common marker of stress, but its concentration depends on many factors such as physical or psychological stress, environmental conditions and metabolic factors, giving way to a high inter- and intra-individual variation [33]. Moreover, the sampling of this substrate generates a substantial stress response by itself in the animals [34]. Non-invasive and stress-free substrate such as saliva have also been tested, but the low protein concentration of this substrate, the inherent variability and experimental artefacts during sample preparation and analysis are some of the main limitations [35, 36]. On the other hand, have been reported that psychophysical stress like the one caused by animal transport can induce the response of acute phase proteins (APPs) [37]. The APPs are a group of blood proteins (e.g. haptoglobin, serum amyloid A) mainly synthesised in the liver that have been shown to increase in concentration following stress and so may be useful for the animal welfare assessment [38].

In this study, to overcome some of this limitation we focussed on the identification of transport stress biomarkers on a trusted substrate collected *post mortem* from porcine muscle exudate, collected following centrifugation of muscle specimens [14, 39, 40].

To our knowledge, this work is the first study that applies a label-free method to unravel the proteome differences in transport stress in pigs. Previous studies investigating proteomics changes on stress in pigs used mainly gel-based proteomics, allowing the characterisation of only few proteins/spots [12, 35]. A method like label-free LC-MS helps to overcome many limitations encountered in 2DE (analysis of low or high molecular weights proteins, membrane, very hydrophobic proteins) [41] by enabling the detection of a greater range of acidic, basic and hydrophobic proteins simultaneously [42, 43]. Therefore, we assumed that this method would allow us to identify a more exhaustive list of stress biomarkers.

When cells in response to stimuli like stress are perturbed, the expression levels of some specific proteins are changed to face their new requirements and adjust their cellular functions accordingly [44]. The expression level of proteins depend on the balance between the rate of their synthesis and degradation, the protein turnover which is controlled by the proteostasis [45]. The sum of several consecutive steps that are going from transcription to protein folding will give the birth of a new proteins [46], whereas the protein degradation is responsible for the removal of misfolded, aged or damaged proteins [47]. Complex systems of surveillance monitor and regulate the protein turnover. The growing new polypeptide chains are under the control of molecular chaperones, insertases and translocases, whereas the protein degradation occurs thanks to the lysosome and the proteasome activities [44, 48–51]. One major drawback of biochemical approaches is the assessment of the short-term fluctuations in protein turnover [52]. The synthesis of protein from mRNA, translation is taking place in the cytoplasm. Thanks to the substrate used in our study, an exudate collected from the muscle following centrifugation (with is rich in proteins from the cytoplasm that are easier to extract by centrifugation) and thanks to the advancement in the field of mass spectrometry, was possible to detect low-abundant proteins at short time points and proteins involved in the monitoring of the protein turnover [44].

In our study, the label-free LC-MS analysis revealed a total of 1,464 proteins across all groups, which were involved mainly in cellular and metabolic processes, biological regulation and localisation. Following LC-MS/MS data analysis with Progenesis QI for Proteomics, 66 proteins were found to vary significantly in abundance in response to transport stress (same genetic line after short or long road transport). Some of these proteins (e.g. creatine kinase, glyceraldehyde 3-phosphate dehydrogenase, apolipoprotein) were also identified in other studies carried out in pigs in response to stressors [53–55]. Proteins involved in the regulation and monitoring of the proteins turnover were identified (e.g. proteasome). Most of the identified proteins were involved in several cellular and metabolic biological process, in blood coagulation and glycolysis related pathways that characterise the stress response. These results are in agreement with classical methods used to evaluate the stress response. For example, measures of creatine kinase (CK) in plasma is often used to reflect stress coping characteristics and metabolic status of the animal as it is reported to change in response to stressors [55, 56]. Changes of the environment and subsequent homeostatic adaptation are responsible for different genetic expressions but it is well known that these perturbations are not the same for the modifications in the proteome.

This study highlight several proteins, like blood plasma proteins, heat stress proteins, lipidic proteins which may activate pathways related to inflammatory response, to coagulation, to complement pathways, etc. For example, several blood plasma proteins were identified, these proteins play a key role in immune-defense against pathogens, in the energy metabolism regulation, hormone transport and blood clotting [57]. Plasma proteins are often used as markers of the physiological state of an individual as their levels reflects the level of homeostasis [58]. Among the proteins present in the plasma, APPs are species-specific and change in concentration (positive if they increase their synthesis or negative if it decrease) in response to stimuli like infections, stress, etc. [59, 60]. Studies conducted on pigs [37, 61, 62] and cattle [63, 64] showed that APPs increase in abundance after long distance transportation. Several plasma proteins were identified in our study (e.g. complement proteins, plasminogen), which were up-regulated in the non-stressed animals. It have been shown that the higher concentration of APPs in the serum is reached within 24 to 48 h after the stimuli [65, 66]. Studies on pig stress found an increment in APPs after 6 h of transport [37, 67]. Thus, our results are in agreement with these studies, indeed, the down-regulation of the plasma proteins observed in our study in the stressed animals, probably reflect the shorter time that the animals were expose to stress (transport for about 3 hours), higher values are likely to be obtained if the transport of the animals was longer.

Several proteasome and heat shock proteins were characterised among the highlighted proteins. These proteins in response to stimuli like stress, mediated protein refolding or are involved in protein degradation as self protection of the cells.

Indeed, PPI analysis of the highlighted proteins showed distinct clustering of six proteasome proteins. According to their functions, this may contribute to explain in part the difference in stress response. Proteasomes are found in the cytoplasm and nucleus, and also degrade proteins in the endoplasmic reticulum. They are crucial elements of the ubiquitin-proteasome system (UPS), which is involved in many biological processes, such as cell cycle, cell differentiation and metabolic adaptation. It has been shown that proteasomes inhibition leads to the accumulation of non-degraded proteins that are potentially toxic and can lead to an amino acid imbalance. Therefore, cells need to increase proteasomes abundance in order to survive in stress conditions that increase the demand for protein degradation, such as increased levels of misfolded proteins [68]. Environmental stresses are manifold and so the stress response can therefore differ greatly among tissues and cell types. In this processes, the UPS plays a pivotal role in counteracting the effects of stressors [69]. Herrmann et al. [70] highlighted the importance of proteasomes function in a healthy heart in pigs. They demonstrate that chronic proteasomes inhibition would affect the cardiovascular system, leading to the structure and the function alteration of the heart. A transcriptomic study of Li et al. [71] investigated the global gene response to chronic heat stress exposure in broiler. A total of 110 genes were detected to be differentially expressed in breast tissue involved in different pathways such as the ubiquitin-proteasome. Their findings confirmed that the ubiquitin-proteasome pathways are involved in heat regulation. In the current study, the increased abundance in the animals subject to long road transportation may allude to a role of these proteins as markers for testing the level of stress response in pigs subject to road transportation. Thus, to survive in stress conditions that increase the demand for protein degradation, such as increased levels of misfolded proteins, proteasomes abundance are increased by the cells.

Heat shock proteins (HSPs) are a family of conserved molecular chaperones, and their role is to protect, preserve or recover the proper functional conformation of proteins [72]. Only a small amount of HSPs are constitutively expressed, a huge amount are synthesised upon stimuli such as growth, development and differentiation and in response to stresses (e.g. heat, exercise, transport) [73, 74]. During stresses, damaged proteins are either repaired by HSPs or the UPS for protein degradation, thereby avoiding the accumulation of cytotoxic contents [75]. HSPs mediated protein protection and cell signalling, as well as UPS degradation are thus central to cellular homeostasis, and are reported to play substantial roles in stimuli resistance. HSP90 and HSP70 are the two most important HSPs that interact with the denatured proteins to help their refolding and reassembling, so that proteins can turn back to their active forms [76]. STIP1 is a 62.6-kDa protein also known as HSP-organizing protein that functions as an adapter that directs HSP90 to HSP70 client protein complexes in the cytoplasm, ultimately modulating their chaperone activity. In our study, two HSPs (HSPA8 and STIP1) were identified with a higher abundance in pigs subject to long road transportation. Not many proteomics studies have investigated the influence of pre-slaughter handling practices like animal transportation. Using ELISA, Yu et al. [13] investigated the effect of transportation on the expression of four HSPs (alpha-B-crystalline, Hsp27, Hsp70 and Hsp90) in the *longissimus dorsi* of pigs, observing a decrement of the expression of these proteins after transportation. Similar results were also obtained by Bao et al. in response to transport stress using the same substrate [77] and in pig hearts [78]. It is of interest to note that in contrast with the data mentioned above, the two stress related proteins highlighted in our study show a higher abundance in pigs subject to long road transportation. This is probably due to changes in solubilisation of the proteins in our substrate (exudate collected following centrifugation) [79, 80]. The differential localisation of the protein in relation to cellular stress could explain the higher abundance of this protein in our substrate, which may allude to a weaker activity of HSPs after transport stress.

Cellular homeostasis requires a balance between HSPs and UPS, with the unfolded or damaged proteins either repaired by HSPs immediately or degraded by proteasome pathway as is the case in our study.

Cellular redox balance can be challenged by environmental stress, leading to an increased production of reactive oxygen species (ROS), inducing to the accumulation of various harmful metabolites and eventually cause serious damage to DNA, proteins and lipids [81, 82]. To withstand these adverse processes, cells have developed protective systems that either repair the damage or destroy the reactive oxygen species. Superoxide dismutases control the reduction of the superoxide, but in turn produce hydrogen peroxide [83]. Catalase is an antioxidant enzymes that mitigates oxidative stress and play an important role in the elimination of hydrogen peroxide and promote its catalysis in water and oxygen. Deficiency of this enzyme have been postulated to be related with the pathogenesis of many age associated degenerative diseases like Alzheimer and Parkinson’s diseases, dermatological disorders, etc. [84]. A study of Selsby [85] assume that catalase overexpression improve the resistance to fatigue and reduced contraction induced injury in dystrophic skeletal muscle. This data are in line with what we observed in our study, an overexpression of catalase in stressed animal. Indeed, the ROS formation during the stress caused by the transport require specific adaptations, such as an increased activity of the enzymes repairing the oxidative damage, an increased resistance to oxidative stress and lower levels of oxidative damage.

This label-free quantitative proteomics study is a first pilot study applying this technology to transport stress in pigs, expanding the proteome coverage of the substrate used (muscle exudate), providing new insights into pigs self-regulation processes under transport stress and enhancing the characterisation of this species that is considered a suitable model for several biomedical aspects. 66 proteins were up- or down-regulated in muscle exudate in relation to transport stress using label-free LC-MS proteomics. These proteins were mainly involved in cellular process, metabolic process and in response to stimulus and could be involved in the biochemical process involved in transport stress. Catalase and STIP1 were further confirmed by Western blot. In the events of stresses, damaged proteins are either repaired by HSPs or UPS for protein degradation. Our results contribute to a broader understanding of the adaptability to transport stress in pigs. Moreover, several biomarkers that can be potentially useful to define new molecular phenotypes for novel applications in genetic selection and breeding for stress resistance were highlighted.

## Supporting information

S1 Table Full list of the 1464 proteins identified by mass spectrometry in the swine muscle exudate in the non-stressed and stressed pigs determined in Proteome Discoverer using SEQUEST HT algorithm. MS files were searched against *Sus Scrofa* protein database from UniProt (1,428 reviewed proteins and 47,760 unreviewed TrEMBL) downloaded April 2019. ^a)^The total number of identified peptide sequences (peptide spectrum matches) for the protein, including those redundantly identified. ^b)^The number of peptide sequences unique to a protein group. ^c)^Theoretical molecular weight.
(XLSX)

S1 Fig Percentage of the 66 proteins identified with altered levels in response to transport stress (stressed) compared to short road transportation (non-stressed) (Table 1) grouped according to different pathways (PANTHER). (TIF)

S1 Raw images Raw Western blot images of stress induced phosphoprotein 1 (STIP1) and catalase in pig muscle exudate and raw images of the membranes stained using the reversible stain Ponceau S. Numbers (1 to 8) between the image of the membrane and the image of the Western blot indicate the eight animals used in the experiment for each pig (1 to 4: non-stressed pigs; 5 to 8: stressed pigs), each of which was run in an individual gel lane. The Western blot (using the same animals) was repeated three times [three technical replicates (A, B; C)]. The image of the membranes stained using the reversible stain Ponceau S were used to normalise the value of the Western blot images and used for statistical analysis.
(PDF)
