# Supplementary figures and images for: Label-free quantitative proteomics and stress responses in pigs—The case of short or long road transportation
Source: PLoS One. 2022 Nov 23;17(11):e0277950. doi: 10.1371/journal.pone.0277950 (PMC9683611; doi:10.1371/journal.pone.0277950)

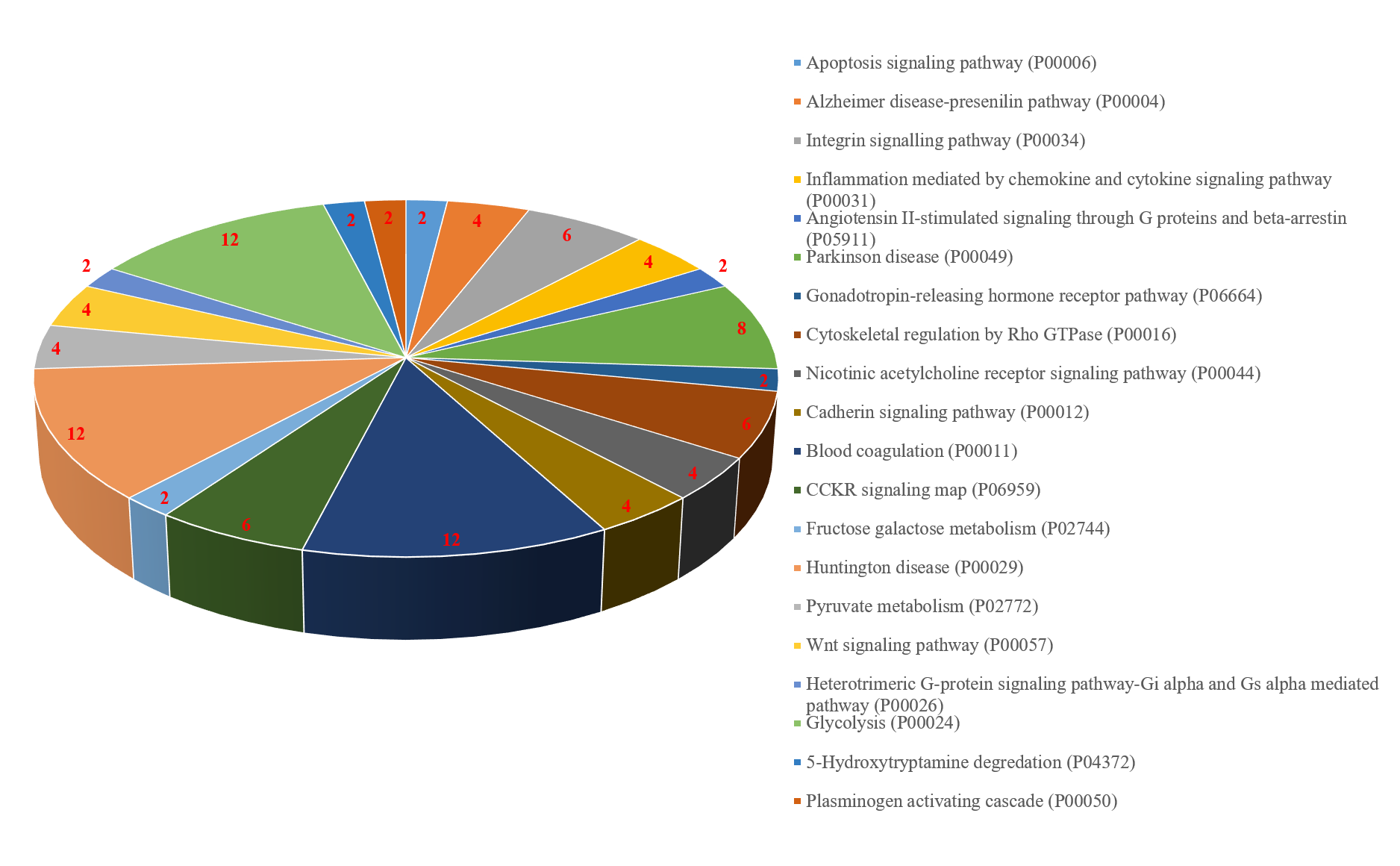

Supplement: S1 Fig — (TIF) [file pone.0277950.s002.tif]
