# Supplementary material for: Label-free quantitative proteomics and stress responses in pigs—The case of short or long road transportation
Source: PLoS One. 2022 Nov 23;17(11):e0277950. doi: 10.1371/journal.pone.0277950 (PMC9683611; doi:10.1371/journal.pone.0277950)

Stress induced phosphoprotein 1 (STIP1)

Technical replicates (A, B, C):

- X: Samples not used
- 1 to 4: non-stressed pigs
- 5 to 8: stressed pigs

Membranes stained using the reversible stain

Western blot STIP1

A

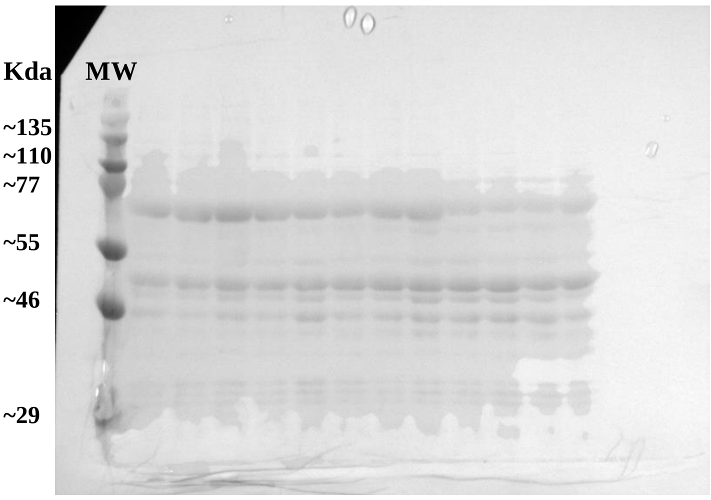

B

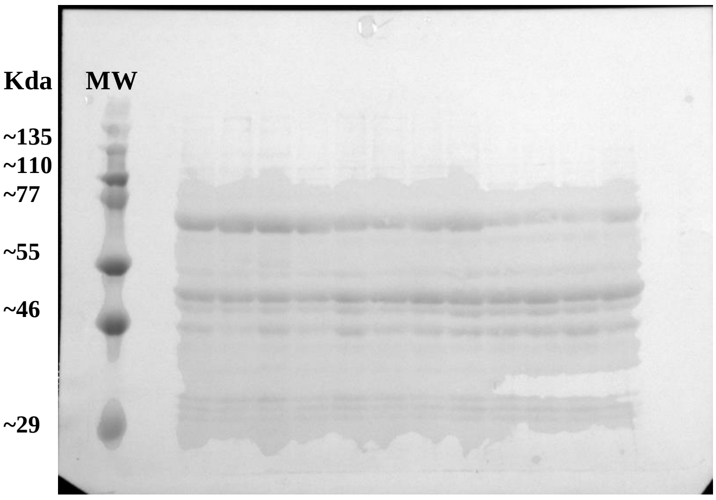

C

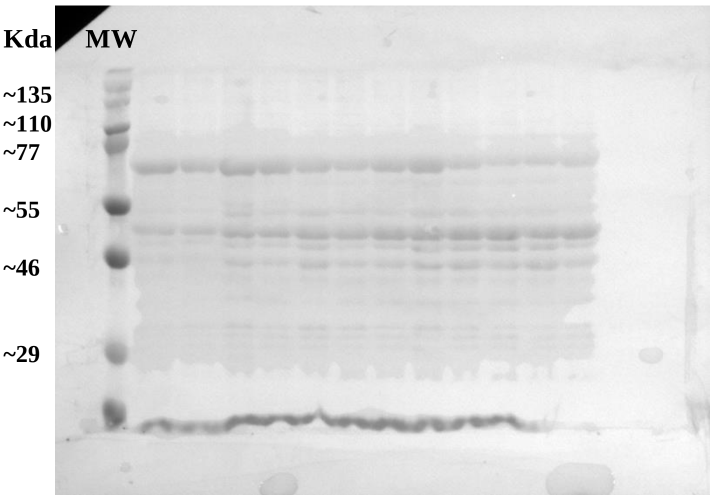

Catalase (CAT)

Technical replicates (A, B, C):

- X: Samples not used
- 1 to 4: non-stressed pigs
- 5 to 8: stressed pigs

Membranes stained using the reversible stain

Western blot CAT

A

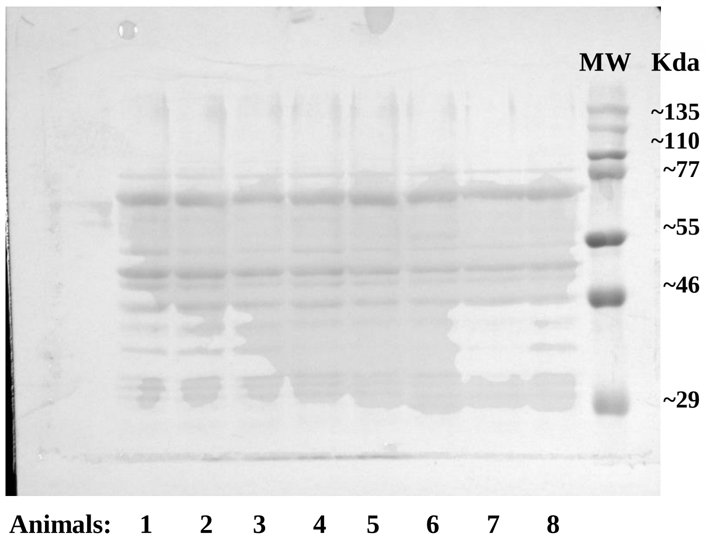

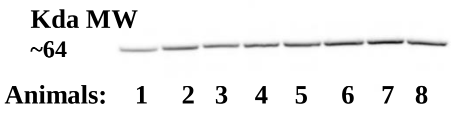

B

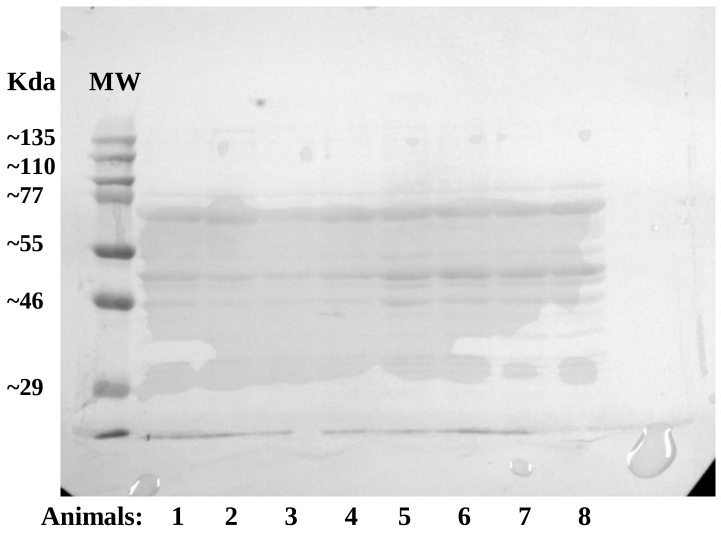

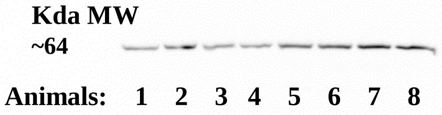

C

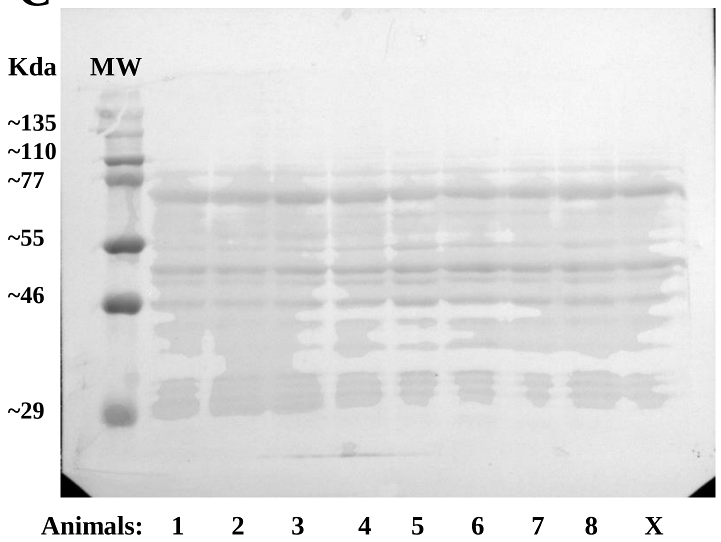

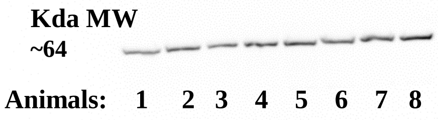

Supplement: S1 Raw images — Numbers (1 to 8) between the image of the membrane and the image of the Western blot indicate the eight animals used in the experiment for each pig (1 to 4: non-stressed pigs; 5 to 8: stressed pigs), each of which was run in an individual gel lane. The Western blot (using the same animals) was repeated three times [three technical replicates (A, B; C)]. The image of the membranes stained using the reversible stain Ponceau S were used to normalise the value of the Western blot images and used for statistical analysis. (PDF) [file pone.0277950.s003.pdf]
